# Supplementary material for: Electronic health record and genome-wide genetic data in Generation Scotland participants
Source: Wellcome Open Res. 2017 Sep 18;2:85. [Version 1] doi: 10.12688/wellcomeopenres.12600.1 (PMC5645708; doi:10.12688/wellcomeopenres.12600.1)
Supplement: Peer review history

# Peer review: Referee response for version 1

**DOI:** 10.21956/wellcomeopenres.13644.r26066

**Published:** 2017-10-16

**Authors:** Susan J. Lindsay

**Affiliations:** 1 Institute of Genetic Medicine, Newcastle University, Newcastle upon Tyne, UK

This paper describes linkage of electronic health records to an already well-described genetic and phenotyping resource access to which has proved instrumental in many studies. The strong case for the research advantages of linking electronic health records is made very clearly and interesting examples selected to illustrate the arguments. The paper also addresses important areas that are potential concerns or limitations; for example evidence for the quality of the NHS data and approaches to making data that has been collected in different locations and time-periods comparable are well-described. As well as referring to earlier papers, much of the supporting information on the participant cohort or needed to assess the procedures used and organisation and labelling of the datafiles are briefly referred to in the paper with the detail necessary available via links to other websites. Given the scale and complexity of the resource, I think this is acceptable. The Generation Scotland website, for example, describes access policy, including mechanisms for ensuring data security. Data security measures are also described on the Safe Haven websites. The ethical framework, is outlined in the Ethical Statement and Consent paragraphs and also addressed in several places on the GS website, including in the access policy, and specifically relating to participant confidentiality.

 Embedded links to external sites show the excellent informatics and other support context for the GS programme. Indeed, more generally, the paper would be a very good starting point for readers wanting to find out about the field of eHealth research.    

 In research terms, the paper illustrates that the vision of the co-founders and the work of the GS programme have borne fruit in many ways. Linking the NHS Scotland EHR hugely extends the research potential of the resource and should contribute to “closing the loop” i.e. new treatments and therapies for NHS patients (and patients outside the UK) in the incoming years.

I have read this submission. I believe that I have an appropriate level of expertise to confirm that it is of an acceptable scientific standard.

# Peer review: Referee response for version 1

**DOI:** 10.21956/wellcomeopenres.13644.r26065

**Published:** 2017-10-11

**Authors:** Carol Brayne

**Affiliations:** 1 Cambridge Institute of Public Health, University of Cambridge, Cambridge, UK

This paper describes the way in which data linkage has been implemented for Generation Scotland. This is a volunteer cohort of adults with extensive research data including imaging, genomic, -omic and other phenotyping for whom consent was asked for linkage to different types of data available from other sources, most particularly health service records. This builds on the outstanding opportunities that exist in Scotland which has suffered less from fragmentation and barriers to access to appropriate routine data than particularly England. Most provided consent so that there is a rich set of data, being continuously updated but which researchers can seek approval to access at any one time for approved research. The paper describes the data linkage itself in detail, outlining the nature of the datasets and the processes. This is, in of itself, valuable for cohorts elsewhere as these are areas that have been extremely challenging to pursue, even when consent from individuals is present.

 Data are available for a decade on these individuals.  Much of the data to fully understand the cohort is available in other publications, most notably the International Journal of Epidemiology cohort series. The reader cannot establish from this paper what the provenance of the cohort is in terms of its relationship to the source Scottish population (response rate, known response biases etc) but this is not the purpose of the paper. Perhaps it should not be called a Family Health Study given its likely response biases and the fact it is very unlikely to be fully representative of Scotlands families, but this is more important for the detail of what kind of research these rich data lend themselves to and what they would not be so well suited to. 

 The description of how to access looks very good, but it is in the doing that people find out what it’s really like. It might be helpful to consider having a flow chart to illustrate the process, and perhaps have some case examples (even if only referred to on line) for different types of access taking the reader through the steps for different types of work.

 Some minor comments: data are plural; what is the detail of neonatal inpatient information; some of the diagrams about actual data could be more informative; relatedness is discussed too briefly; there is no discussion of limitations.

I have read this submission. I believe that I have an appropriate level of expertise to confirm that it is of an acceptable scientific standard, however I have significant reservations, as outlined above.

# Peer review: Referee response for version 1

**DOI:** 10.21956/wellcomeopenres.13644.r26079

**Published:** 2017-10-02

**Authors:** Sara J. Brown

**Affiliations:** 1 Skin Research Group, School of Medicine, University of Dundee , Dundee, UK

Thank you for asking me to review this Data Note describing the valuable resource created through linkage of electronic health records to research data in the Generation Scotland Scottish Family Health Study.

 I answered ‘partly’ to question 3: *Are sufficient details of methods and materials provided to allow replication by others?* because it would be useful to have more detail regarding the inclusion/exclusion criteria for the individuals chosen for participation & selected for genotyping. It would also be helpful to clarify how issues such as changes in the normal range definition of biochemical assays are addressed in these longitudinal datasets?

 I answered ‘partly’ to question 4: *Are the datasets clearly presented in a useable and accessible format?* because it would be useful to have:

 (a) clarification in the abstract which states ‘the 1 ^st^ detailed demonstration of the research value….’ although 3 publications have appeared to date.

 (b) clarification in the abstract regarding availability/cost/ethical considerations of accessing the data.

 (c) clarification in the abstract of the terms ‘registrations, attendances, samples’ in this context.

 (d) a section in the main manuscript describing the real-word limitations, perhaps listed in a box or table alongside the many strengths that are already emphasized. It would also be very helpful to compare/contrast the strengths of GS:SFHS alongside other population genetics research resources including UK Biobank and eMERGE.

 (e) Figure 1 is nice; please consider adding the number of individuals for whom selection criteria were not met; spelling out the abbreviations in the NHS cylinders; adding to the flow-chart to show how approval is obtained.

 (f) the manuscript mentions the availability of environmental data eg smoking but this data is not described further.

 (g) the example of urate is useful, however the statement that this is 27 ^th^ in the top 30 and yet shows a significant result in GWAS reflects in part the strength of a single-effect locus on urate levels so it is not appropriate to use this as a justification for the overall study size.

 (h) the statement on data availability ‘…GS:SFHS cohort is available to researchers in the UK and to international collaborators…’ is unclear. Are international groups only able to access the data if they have UK collaborators?

 (i) more detail on the future plans for this collection to grow/mature as mentioned in the final paragraph.

 I have some minor suggestions to improve the report and its formatting:

 (i) The methods stating DNA extraction & genotyping are given before the subset selection; it would be more logical for this information to be first.

 (ii) The subtitle ‘Dataset validation’ does not describe what appears in subsequent paragraphs; please add more sub-headings that more correctly match the methods.

 (iii) The legend in Fig 2 says the ‘exact numbers’ are shown, but these are for the dark blue bars not the light blue data – please clarify.

 (iv) please replace abbreviations/acronyms in Fig 2 with words.

 (v) please replace light blue bars replaced with a more vivid colour for visibility.

I have read this submission. I believe that I have an appropriate level of expertise to confirm that it is of an acceptable scientific standard.
